# Direct Detection of Unnatural DNA Nucleotides dNaM and d5SICS using the MspA Nanopore

**DOI:** 10.1371/journal.pone.0143253

**Published:** 2015-11-20

**Authors:** Jonathan M. Craig, Andrew H. Laszlo, Ian M. Derrington, Brian C. Ross, Henry Brinkerhoff, Ian C. Nova, Kenji Doering, Benjamin I. Tickman, Mark T. Svet, Jens H. Gundlach

**Affiliations:** Department of Physics, University of Washington, Seattle, Washington, United States of America; The Scripps Research Institute, UNITED STATES

## Abstract

Malyshev *et al*. showed that the four-letter genetic code within a living organism could be expanded to include the unnatural DNA bases dNaM and d5SICS. However, verification and detection of these unnatural bases in DNA requires new sequencing techniques. Here we provide proof of concept detection of dNaM and d5SICS in DNA oligomers via nanopore sequencing using the nanopore MspA. We find that both phi29 DNA polymerase and Hel308 helicase are capable of controlling the motion of DNA containing dNaM and d5SICS through the pore and that single reads are sufficient to detect the presence and location of dNaM and d5SICS within single molecules.

## Introduction

**Fig 1:**
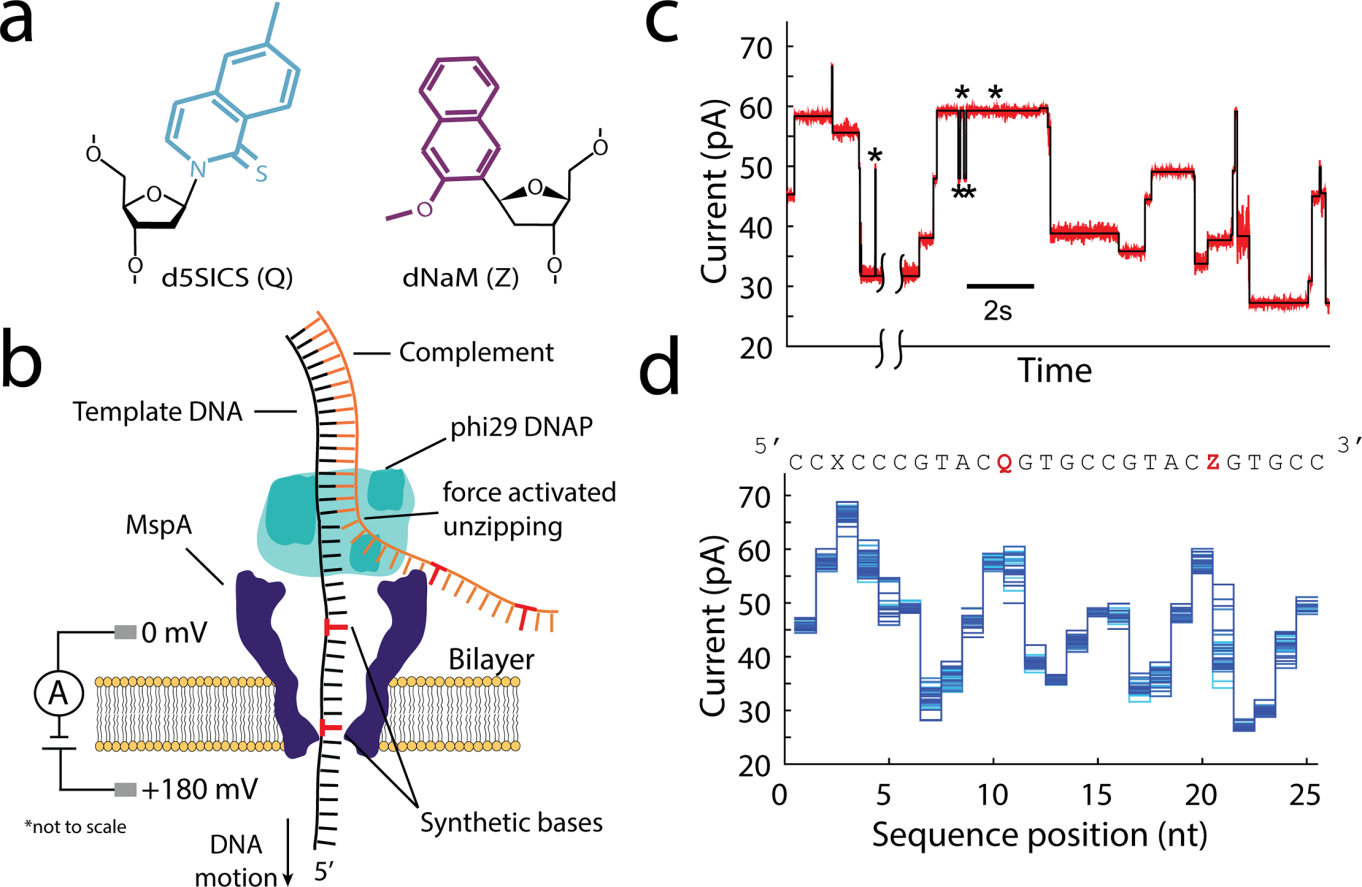
Unnatural DNA nucleotides & nanopore sequencing. **(a)** Chemical structures of the unnatural nucleotides d5SICS and dNaM. **(b)** An illustration of the nanopore system. The voltage draws the DNA into the pore, with the polymerase coming to rest on the rim of MspA. The force by the electric field draws the DNA into the pore, thereby dissociating the dsDNA in single nucleotide steps; the DNA moves against the direction that polymerase would move while synthesizing dsDNA. The nucleotides positioned in the narrowest section of the pore (the constriction) modulate the current through the pore. **(c)** An example current trace in red taken with the phi29 DNAP. Automatically detected discrete current levels are displayed in black. Each level corresponds to a single nucleotide movement of the DNA through MspA. Asterisks indicate repeated levels caused by backwards motion of the DNA. The time-axis is broken for a level longer than 10 seconds in duration. **(d)** The consensus of time-ordered ion current level amplitudes for the sequence printed above, compiled by collecting 41 current traces like those in (c), removing the temporal information, and aligning them to an ion current reference. The unnatural bases dNaM and d5SICS are denoted by ‘Z’ and ‘Q,’ respectively, and emphasized by red text. An abasic residue is indicated by ‘X.’

Nature uses the four natural nucleotides, A, C, G and T, to encode genetic information. However, *Escherichia coli* was recently shown to be capable of propagating an expanded genetic alphabet that includes the unnatural nucleotides dNaM and d5SICS (Fig 1A) [1]. Since mainstream DNA sequencing methods only sequence the standard nucleotides, Malyshev *et al*. identified incorporation of d5SICS and dNaM by an abrupt termination of the Sanger sequencing fluorescence signal [1]. Yang *et al*. have devised a method for sequencing a 6-letter genetic alphabet that relied on mutation of the unnatural bases back to natural bases followed by traditional sequencing methods (although the bases sequenced were not dNaM and d5SICS) [2]. Such indirect methods of detecting unnatural bases are not ideal. For instance, the Sanger sequencing termination method only shows that an unnatural nucleotide is present in the sequence and cannot presently differentiate between unnatural bases or detect multiple unnatural nucleotides along the same strand. Chemical conversion methods add complication and potential for error. Identification of non-standard nucleotides carried in nucleic acids requires a sequencing platform that is capable of detecting them directly. Nanopore sequencing is an emerging single-molecule DNA sequencing technology that can meet these requirements [3].

In nanopore sequencing, a nanometer sized pore in a thin membrane forms the only electrical connection between two salt solutions. A voltage applied across the pore causes an ion current to flow through the pore. Negatively charged single-stranded DNA (ssDNA) is drawn through the nanopore by the electric field. The nucleotides in the pore block the ion current flowing through the pore to characteristic levels, allowing determination of the base sequence. To realize this system in practice, we establish a phospholipid bilayer across a ~ 20 μm diameter Teflon aperture, which is immersed in ~ 60 μl of a buffered KCl solution ([4–7]). Protein nanopores insert spontaneously into these bilayers. A single channel is recognized by a sudden, characteristic increase in the conductance. The nanopore with the best proven base distinction is the protein pore *Mycobacterium smegmatis* porin A (MspA, Fig 1B) [5, 6]. MspA is an octomeric channel protein, whose constriction is 1.2 nm in diameter, and 0.6 nm in height. These dimensions are similar to those of ssDNA nucleotides, making MspA a probe that is highly sensitive to the DNA nucleotides in the constriction. ssDNA passes through nanopores too quickly to be read but its motion through the nanopore can be controlled by using enzymes such as the phi29 DNA polymerase (DNAP) [8, 9] or the Hel308 helicase [7]. Nanopore sequencing with MspA has been shown to be sensitive to each of the four canonical nucleotides at the single molecule level [8] as well as epigenetic modifications such as 5-methylcytosine, 5-hydroxymethylcytosine [10, 11] and other cytosine variants [12], which makes MspA a promising candidate for the detection of non-standard nucleotides.

Here we show that the phi29 DNAP can be used in a forced unzipping mode [8, 9] to move DNA containing dNaM and d5SICS through MspA. In this method, the phi29 DNAP binds to the template strand at a frayed 5′-3′ junction where it cannot perform any polymerase function. The 5′ end of the template is drawn into the pore until the enzyme comes to rest on the rim of MspA. The electrostatic force pulls on the DNA template strand, and the phi29 DNAP acts as a wedge, unzipping the double-stranded DNA (dsDNA) one base at a time (Fig 1B). This results in ion current traces containing distinct, millisecond time scale current levels (Fig 1C). The raw data is reduced to a sequence of current-level amplitudes, which are reproducible to the picoampere scale (Fig 1D). By aligning the measured current levels to the known DNA sequence, these ion current sequences are combined to create a consensus of current levels [8, 11, 13]

## Results

**Fig 2:**
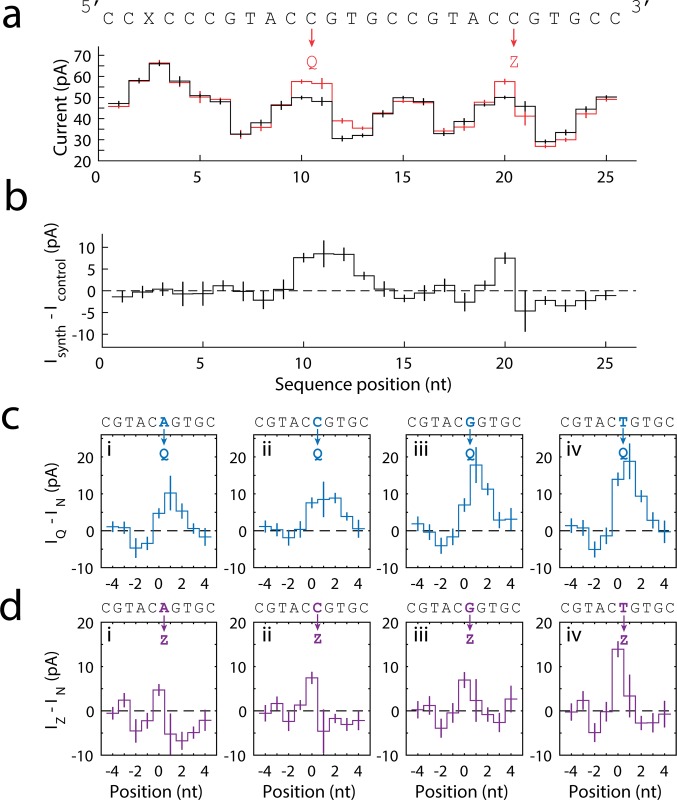
Analysis of ion currents. **(a)** Consensus of current levels for a DNA strand containing one instance of the nonstandard nucleotides dNaM and d5SICS (red, N = 41 events), and for control sequence A containing cytosines in place of dNaM and d5SICS (black, N = 19). The error bars represent the standard deviation of the measured current levels. **(b)** The difference of the current level patterns from (a), obtained by subtracting the black curve from the red curve. The nonstandard nucleotides cause a significant current difference in their immediate vicinity. **(c)** The current difference caused by substituting d5SICS in place of natural nucleotides. The four panels correspond to the substitutions Q for A (i), Q for C (ii), Q for G (iii), Q for T (iv). The location of the substitution is emphasized by blue text in the sequence displayed above, and is referred to as position 0. **(d)** The current difference caused by substituting dNaM for the natural nucleotides. The four panels correspond to the substitutions Z for A (i), Z for C (ii), Z for G (iii), Z for T (iv). The location of the substitution is emphasized by purple text in the sequence displayed above, and is referred to as position 0. All error bars are the standard error.

We performed nanopore sequencing with DNA constructs containing one instance of each of the unnatural bases dNaM and d5SICS. By comparing reads to control sequences (control sequences A, B, and C, S1 Table) that contained natural nucleotides in the positions of the unnatural bases, we were able to determine how distinguishable the unnatural bases were from the canonical bases. In Fig 2A we plot the consensus of current levels for the strand containing the unnatural nucleotides and the consensus read for control sequence A. These two sequences were identical, except that two cytosines were replaced with dNaM and d5SICS. The current level sequences reveal significant current differences for about four levels near the positions of each substitution. Fig 2B shows the difference of the two current level plots to isolate the effects of the substitutions (see also S1 Fig). The current difference persists over a range of about four nucleotides, consistent with previous measurements involving single-nucleotide polymorphisms [6, 8] and methylated vs unmethylated nucleotides [10–12]. The error bars are the standard deviations of the individual reads, showing that single reads are sufficient to differentiate dNaM or d5SICS from cytosine.

To allow for a direct comparison of the ion current change caused by single nucleotide substitutions, we measured the currents for each of the six nucleotides (dNaM, d5SICS, A, C, G and T) in an identical sequence context. As in Fig 2B, we plot the difference in current levels for the replacement of each of the four natural nucleotides with d5SICS (Fig 2C. i-iv) and dNaM (Fig 2D. i-iv). Each substitution produces a significant difference in the current signal. We found that d5SICS consistently causes large increases in current of ~10–20 pA relative to the standard nucleotides. dNaM causes a more complicated change in ion current, with some levels having a larger current and some levels having a smaller current than the standard nucleotides. The large current differences and comparatively small standard deviations show that dNaM and d5SICS are distinguishable from each of the natural nucleotides with a single read.

**Fig 3:**
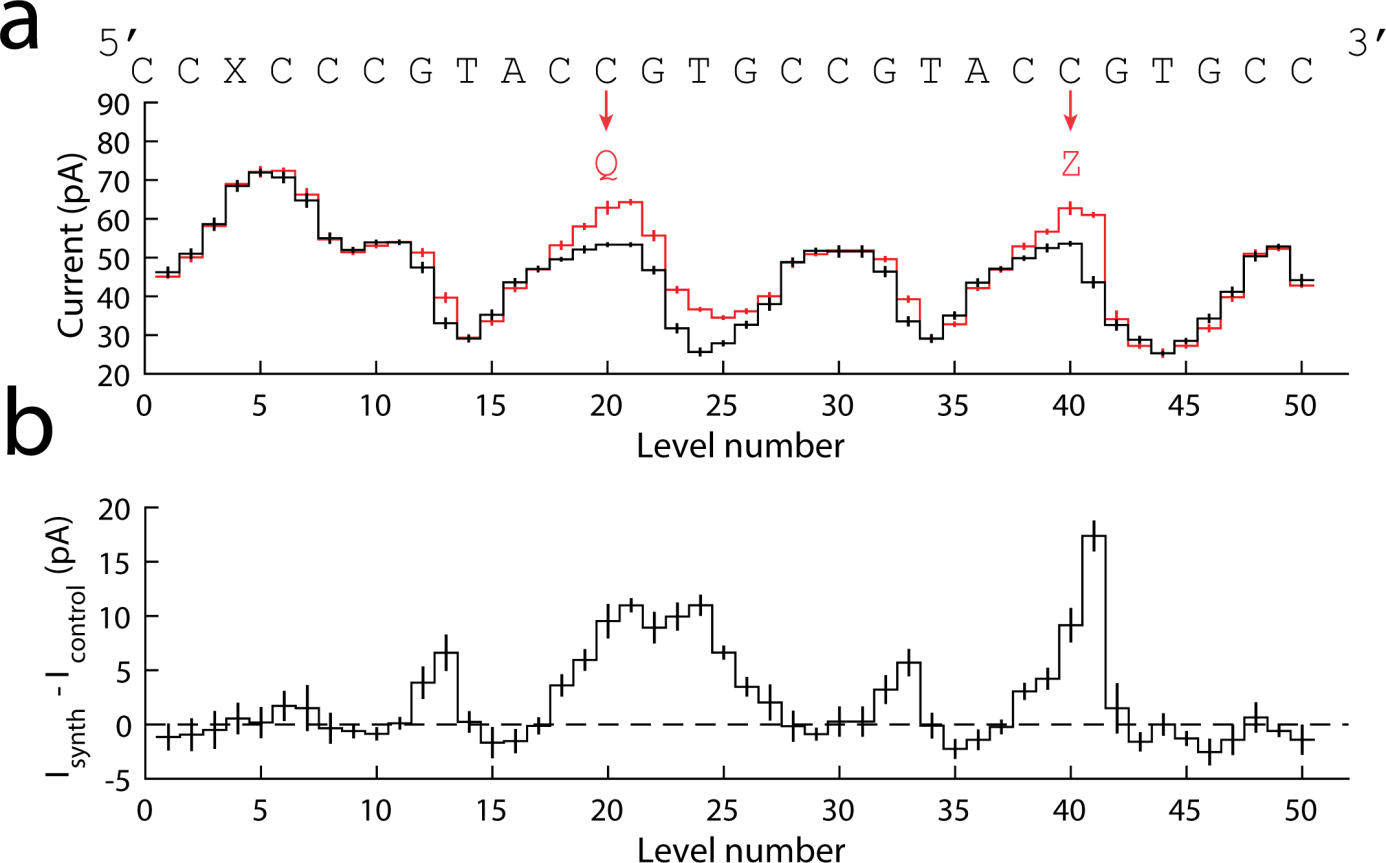
Current levels for Hel308 helicase. **(a)** Consensus of current levels for same sequence as in Fig 2 (red, N = 48 events) and for the same control sequence (black, N = 49 events), but taken with the helicase Hel308 controlling DNA translocation. The levels are plotted in reverse time order so that the sequence is ordered from 5′ to 3′. The current scale is slightly different than in Fig 2A because these data were obtained at different salt conditions. **(b)** The difference of the current level patterns from (a), obtained by subtracting the black curve from the red curve. All error bars are the standard error.

We also examined whether the helicase Hel308 (from *Thermococcus Gammatollerans* EJ3) could process DNA containing dNaM and d5SICS. Hel308 is a 3′ to 5′ superfamily 2 helicase / translocase, which has previously been found to take two distinct steps for each nucleotide translocated [7]. Fig 3A shows the consensus of current levels obtained with Hel308 for the same DNA strands as in Fig 2A. The levels for Hel308 are qualitatively similar to the levels produced by phi29 DNAP, however, Hel308 shows two steps for each progression by one nucleotide. Taking the difference of these curves again reveals a significant difference around the substitution site (Fig 3B). While d5SICS again causes a large increase relative to cytosine, when using Hel308 to control the DNA translocation the difference in current is spread out over approximately nine half-nucleotide steps, as opposed to four full-nucleotide steps with phi29 DNAP. dNaM produces a large increase in current, and does not have the decrease in current present in the phi29 DNAP difference plot (Fig 2B). This could potentially be explained by a high sensitivity of the current on the location of the dNaM nucleotide. MspA has previously been shown to be highly sensitive to DNA positioning within the pore [7]. Current level 21 in Fig 1D takes on a much broader range of current values than is typical for a current level in MspA, indicating that slight changes in the position of the DNA have large effects on the current. This is supported by the abrupt transition between current levels 41 and 42 in Fig 3A.

Unlike in Fig 2B there are significant differences in the current at levels not immediately adjacent to the substitution cite (Fig 3B. levels 12–13, 32–33). These differences could be caused by the unnatural nucleotides inducing a different secondary structure in the DNA, causing a slight repositioning of the DNA in MspA’s constriction, thereby changing the current. These shifts become most evident in areas in which there are large changes in current between adjacent levels, where small changes in DNA position lead to large changes in the current. These differences may not be observed in the phi29 DNAP levels because the current is not being measured in the regions of high current change (Fig 2A. between levels 6–7 and between levels 16–17). These differences are narrower than would be expected for a typical single nucleotide substitution, and are only associated with levels in a high-contrast region, supporting the hypothesis that this is a secondary structural effect.

The second step of Hel308 provides additional information about the base content, and decreases the information loss caused by helicase stepping errors such as skipped levels. Interestingly, Hel308 is capable of walking over the unnatural bases without error. The retained functionality of Hel308 in the presence of unnatural nucleotides, together with the additional information provided by the second step, makes Hel308 a promising enzyme for nanopore sequencing of unnatural bases.

## Discussion

We have shown that the nanopore MspA can be used to directly detect the unnatural nucleotides dNaM and d5SICS within the same DNA strand, and that two enzymes can be used to control the movement of such non-standard DNA through the nanopore. Because the only requirement for nanopore sequencing is that the motor enzyme be able to function on DNA containing the unnatural nucleotides and that the current levels are distinguishable, our results suggest that nanopore sequencing with MspA is generally applicable to the detection of unnatural nucleotides. In particular, nanopore sequencing can be used to verify the success of chemical DNA conversion methods which replace a standard base with an unnatural base. Because of the high current contrasts in MspA, in many cases a single read of a DNA strand with unnatural bases is sufficient to obtain high confidence in the substitution, especially when the substitution site is known.

Given that about 4 nucleotides affect the ion current of each step, *de novo* sequencing of DNA containing d5SICS and dNaM bases in addition to the four natural bases is more complicated. A 6-letter alphabet would translate into 1296 possible current levels, which may leave many current levels indistinguishable. However, further optimizations to nanopore sequencing, such as different mutations to MspA, may increase the separation of current levels, allowing for improved *de novo* sequencing. A full study of dNaM and d5SICS in all possible sequence contexts would have to be carried out to fully assess the possibility of *de novo* sequencing of DNA with such an expanded alphabet.

Our results could help to shed light onto the mechanisms by which bases block the current through the nanopore. Classification of the current patterns for a large number of unnatural bases with varying chemical structures could help to develop models that explain the observed currents in nanopores. For example, d5SICS and dNaM produce larger currents than any of the standard nucleotides, hinting that the ion current may be linked in part to the hydrophobicity of the nucleotides. Improved understanding of the ion current could lead to improved base calling in nanopore sequencing.

## Materials and Methods

### Pore establishment

A single M2-NNN MspA nanopore was established in a 1,2-diphytanoyl-sn-glycerol-3- phosphocholine lipid bilayer using well-described methods[4–8]. Lipids were ordered from Avanti Polar Lipids.

### Operating conditions

All experiments with phi29 DNAP were run with asymmetric salt conditions of 150 mM *cis* KCl and 500 mM *trans* KCl, with 10mM HEPES at pH 8.0, 5 nM DNA, 1 mM EDTA, 1 mM DTT and 10 mM MgCl_2_. Experiments with Hel308 were run with 400 mM *cis* KCl and 400 mM *trans* KCl at 1 mM ATP, with 10 mM HEPES at pH 8.0, 5 nM DNA, 1 mM EDTA, 1 mM DTT and 10 mM MgCl_2_. Experiments were run at room temperature (22 ± 1°C).

### Data acquisition and analysis

Data was acquired at 50 kHz on an Axopatch 200B amplifier at 180 mV and downsampled by averaging to 5 kHz. Ion current levels were selected automatically from raw data using a previously described algorithm[13]. Consensus current sequences were generated using custom software [7].

### Proteins

M2-NNN-MspA (accession number: CAB56052.1), phi29 DNAP (accession number: P03680.1), and Hel308 from *Thermococcus gammatolerans* EJ3 (accession number: WP_015858487.1) were prepared as described in [7].

### DNA design

All DNA was ordered from the PAN Protein and Nucleic Acid facility at Stanford University. dNaM and d5SICS phosphoramidites were synthesized by Glen Research and were incorporated into the DNA oligomers at Stanford using their standard procedure. Template and complement DNA were mixed at a 1:1 molar ratio and denatured at 95°C for 3 minutes, then cooled to 4°C over 10 minutes. All of the sequences used are displayed in S1 Table.

For phi29 DNAP dissociation experiments a complement is annealed to the template DNA strand such that there is a frayed 3′ end on the complement. The phi29 DNAP binds onto the template at the resulting junction. A long 5′ single-stranded DNA tail on the template is drawn into the nanopore, which brings the enzyme to rest on MspA. The force on the DNA dissociates the template from the complement while the phi29 DNAP causes the DNA to move through MspA in single nucleotide steps. Nanopore experiments with phi29 DNAP have also typically annealed a second complement so that when the phi29 DNAP reaches the end of the first complement, it then has the necessary substrate to begin to synthesize double-stranded DNA, resulting in a reversal of the motion of the DNA through the pore [8, 9]. Data for control sequence 2 was acquired with the possibility of synthesis (S1 Table).

For Hel308 translocase experiments, we anneal the complement strand so that there is a free 5′ end, and an 8 base 3′ overhang on the template. The helicase binds at this free end and can begin to unwind dsDNA in solution. The 5′ end of the template is drawn into the pore, dissociating the complement and bringing the helicase to rest on the rim of MspA. Hel308 then walks from 3′ to 5′ on the ssDNA, drawing the ssDNA out of the pore from *trans* to *cis* [7].

DNA sequences 1, 2, and controls A, B, and C (S1 Table) were designed to enable both phi29 DNAP forced unzipping activity and Hel308 translocase activity.

## Supporting Information

S1 Fig dNaM and d5SICS in another sequence context. Consensus of current levels for a DNA strand containing one instance of the nonstandard nucleotides dNaM and d5SICS (red) (N = 48 events), and for control sequence A containing a T instead (black, N = 39). The error bars represent the standard deviation of the measured current levels. **(b)** The difference of the current level patterns from (a), obtained by subtracting the black curve from the red curve. The nonstandard nucleotides cause a significant current difference in their immediate vicinity.
(TIF)

S1 Table DNA strands and Experiment Statistics. A list of all DNA strands and complements used in this study, and the number of events used in the creation of the consensus sequences.
(XLSX)
