# Supplementary figures and images for: Direct Detection of Unnatural DNA Nucleotides dNaM and d5SICS using the MspA Nanopore
Source: PLoS One. 2015 Nov 20;10(11):e0143253. doi: 10.1371/journal.pone.0143253 (PMC4654578; doi:10.1371/journal.pone.0143253)

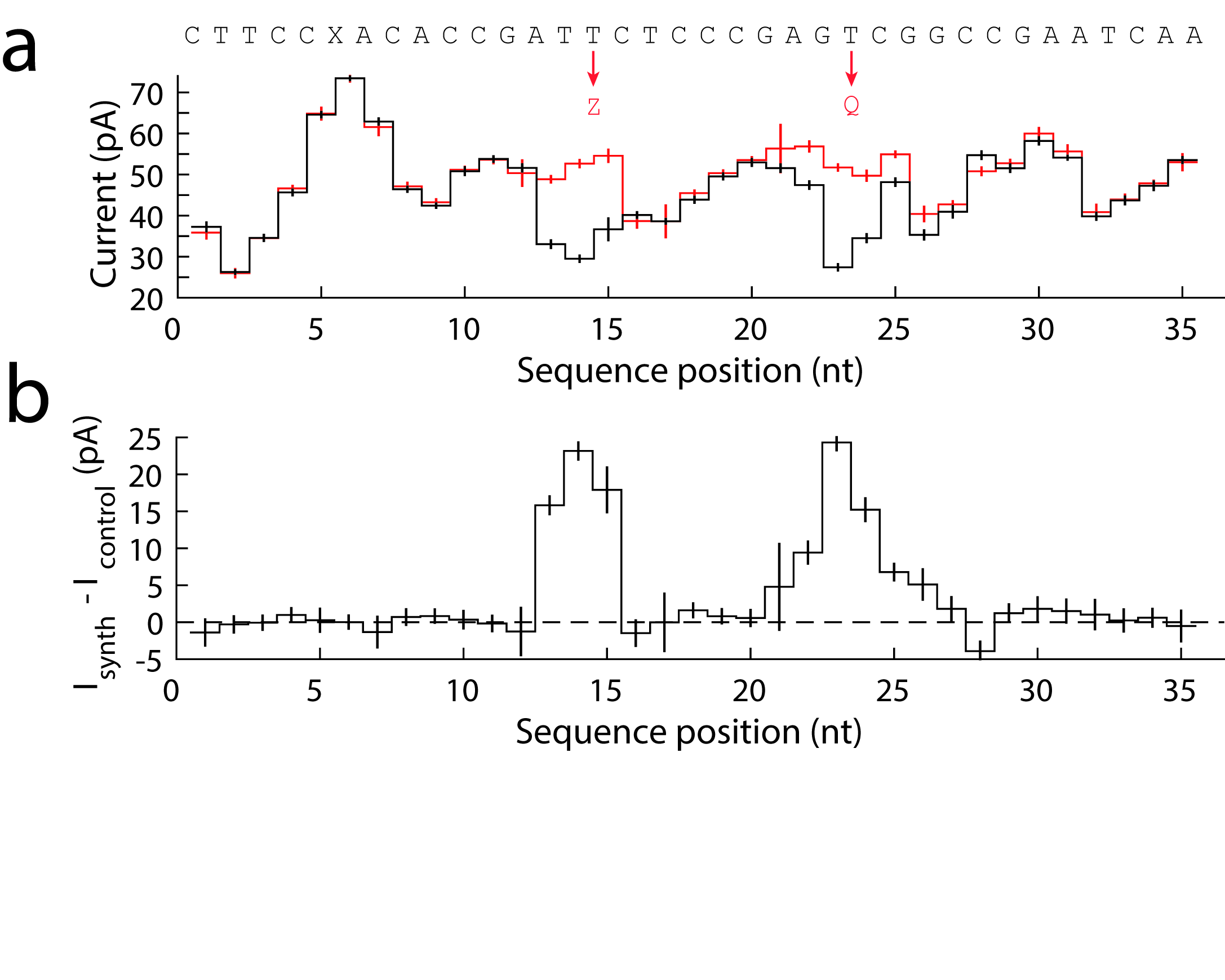

Supplement: S1 Fig — Consensus of current levels for a DNA strand containing one instance of the nonstandard nucleotides dNaM and d5SICS (red) (N = 48 events), and for control sequence A containing a T instead (black, N = 39). The error bars represent the standard deviation of the measured current levels. (b) The difference of the current level patterns from (a), obtained by subtracting the black curve from the red curve. The nonstandard nucleotides cause a significant current difference in their immediate vicinity. (TIF) [file pone.0143253.s001.tif]
